# Vasodilatory Efficacy and Impact of Papaverine on Endothelium in Radial Artery Predilatation for CABG Surgery: in Search for Optimal Concentration

**DOI:** 10.21470/1678-9741-2018-0139

**Published:** 2018

**Authors:** Piotr Węgrzyn, Grzegorz Lis, Paweł Rudzinski, Jacek Piatek, Grazyna Pyka-Fosciak, Ryszard Korbut, Boguslaw Kapelak, Krzysztof Bartus, Radoslaw Litwinowicz

**Affiliations:** 1 Department of Cardiovascular Surgery and Transplantology, John Paul II Hospital, Jagiellonian University Medical College, Krakow, Poland.; 2 Department of Histology, Jagiellonian University Medical College, Krakow, Poland.; 3 Department of Pharmacology, Jagiellonian University Medical College, Krakow, Poland.

**Keywords:** Vasodilation, Coronary Artery Bypass, Radial Artery, Papaverine, Graft Occlusion, Vascular

## Abstract

**Objective:**

The aim of this study was to compare the efficacy of two different papaverine concentrations (0.5 mg/ml and 2 mg/ml) for vasospasm prevention and their impact on endothelium integrity.

**Methods:**

We have studied distal segments of radial arteries obtained by no-touch technique from coronary artery bypass graft (CABG) patients (n=10). The vasodilatory effect of papaverine (concentrations of 0.5 mg/ml and 2 mg/ml) was assessed *in vitro*, in isometric tension studies using *ex vivo* myography (organ bath technique) and arterial rings precontracted with potassium chloride (KCl) and phenylephrine. The impact of papaverine on endothelial integrity was studied by measurement of the percentage of vessel's circumference revealing CD34 endothelial marker.

**Results:**

2 mg/ml papaverine concentration showed stronger vasodilatatory effect than 0.5 mg/ml, but it caused significantly higher endothelial damage. Response to KCl was 7.35±3.33 mN for vessels protected with papaverine 0.5 mg/ml and 2.66±1.96 mN when papaverine in concentration of 2 mg/ml was used. The histological examination revealed a significant difference in the presence of undamaged endothelium between vessels incubated in papaverine 0.5 mg/ml (72.86±9.3%) and 2 mg/ml (50.23±13.42%), *P*=0.002.

**Conclusion:**

Papaverine 2 mg/ml caused the higher endothelial damage. Concentration of 0.5 mg/ml caused better preservation of the endothelial lining.

**Table:** 

Abbreviations, acronyms & symbols				
**ANOVA**	**= Analysis of variance**		**LIMA**	**= Left internal mammary artery**
**BMI**	**= Body mass index**		**NO**	**= Nitric oxide**
**CABG**	**= Coronary artery bypass graft**		**PBS**	**= Phosphate-buffered saline**
**CCS**	**= Canadian Cardiovascular Society**		**PDE**	**= Phosphodiesterase**
**cGMP**	**= Cyclic guanosine monophosphate**		**PE**	**= Phenylephrine**
**CK-MB**	**= Creatine kinase-muscle/brain**		**RA**	**= Radial artery**
**KCl**	**= Potassium chloride**		**RAPCO**	**= Radial Artery Patency and Clinical Outcomes**
**ICU**	**= Intensive care unit**		**RCA**	**= Right coronary artery**
**LAD**	**= Left artery descending**		**SD**	**= Standard deviation**

## INTRODUCTION

Coronary artery bypass graft (CABG) surgery is the most common cardiac surgical procedure that presents long term efficacy and durability with reduced mortality and morbidity observed in the last decade^[1-3]^. For many years, left internal mammary artery (LIMA) has been routinely applied in CABG surgery as "first conduit of choice" because of its resistance to atherosclerosis, especially in older patients^[4]^. Radial artery (RA) is rarely used as a conduit in CABG surgery, mainly because of its high tendency to vasospasm. However, the durability of RA as an arterial conduit is satisfactory: according to Acar et al.^[5]^, patency rates were 93% after 9 months and 89% after 2 years. RA shows higher failure rates when grafted to right coronary artery (RCA) system than to left artery descending (LAD) system. The failures should rather be attributed to the coronary artery than to the RA conduit because of the higher intensity of atherosclerosis in RCA^[6]^.

RA can be applied as a classic conduit or Y-graft, with the proximal site connected with LAD and the distal site grafted into coronary artery. Comparing RA grafts durability with that of veins, RA is much more suitable for CABG: after 5 years of follow-up, the patency of vessels was 98% *vs*. 86%^[7]^. Ferrari and Segesser^[8]^ recommended RA as the "second conduit of choice", after the internal mammary artery, in CABG. Furthermore, it is worth to mention that according to mid-term outcomes in the Radial Artery Patency and Clinical Outcomes (RAPCO) trial^[9]^, there was no essential difference both in patients' free survival time and graft patency time between RA and right internal thoracic artery.

Several techniques are available for bypass graft vessel predilatation during CABG surgery. With an increasing use of RA as a graft, it is very important to understand how the predilatation process can be pharmacologically controlled to improve graft function. We have already compared the vasodilatory effect and impact on endothelium of milrinone 0.4 mg/ml and papaverine 1 mg/ml^[10]^. Our previously research revealed that papaverine in concentration of 1 mg/ml exerts stronger vasodilatory effect on RA and reveals lesser damaging influence on its endothelial cells compared to milrinone 0.4 mg/ml^[10]^. Taking into consideration these results, we continued the study comparing another two different doses of papaverine - two times lower (0.5 mg/ml) and two times higher (2 mg/ml).

The aim of this research was to examine the vasodilatory potential and effect of different concentrations of papaverine on the endothelial integrity in the model of RA segments harvested from CABG patients.

## METHODS

### Patients

We have examined 10 RA segments harvested from patients who underwent CABG surgery. Clinical characteristics as body mass index (BMI), Canadian Cardiovascular Society (CCS) scale, atherosclerosis risk factors, myocardial infarction, and atrial fibrillation were taken into consideration and presented in Table 1. All patients signed the written consent to participate in the study.

**Table 1:** Patients’ clinical characteristics.

Patients’ characteristics		Average ± SD or n (%)
Age (years)		67.5±4.95
Systolic blood pressure (mmHg)		135±7.82
Diastolic blood pressure (mmHg)		86.5±4.84
BMI (kg/m²)		27.51±1.18
CK-MB		38.1±32.56
Hospitalization (days)		8.8±1.93
ICU (days)		2.1±1.2
Post-surgical drainage (ml)		779±469.88
Blood unit transfusion (number)		2.3±1.7
Gender	Male	1 (10%)
	Female	9 (90%)
CCS scale	II	2 (20%)
	III	7 (70%)
	IV	1 (10%)
Smoking		5 (50%)
Arterial hypertension		8 (80%)
Diabetes		7 (70%)
Myocardial infarction		2 (20%)
Atrial fibrillation		7 (70%)
Pressor drugs		10 (100%)
Rethoracotomy		1 (10%)

BMI=body mass index; CCS=Canadian Cardiovascular Society; CK-MB=creatine kinase-muscle/brain; ICU=intensive care unit; SD=standard deviation

### Arterial Rings

Each vessel ring obtained during CABG surgery was cut into six or four smaller rings which were then exposed to organ bath (n=24) and histological examination (n=20).

### Organ Bath

Organ bath experiments were carried out according to standards described previously^[10-13]^. The organ chamber was filled with 5 ml of Krebs-Henseleit buffer, containing 120 mM NaCl, 4.7 mM potassium chloride (KCl), 1.2 mM MgSO_4_, 1.2 mM KH_2_PO4, 2.5 mM CaCl_2_, 25 mM NaHCO_3_, and 5.5 mM glucose, at 37ºC. The rings were mounted between two hooks and were strained to the baseline value of 20 mN. Stabilization to 20 mN was done before each measurement. The rings were precontracted with 20 mM KCl followed by increasing concentrations (10^-9^ to 10^-2^M) of phenylephrine (PE). Next, they were rinsed, stabilized, and immersed in solutions of papaverine (papaverinum hydrochloricum; Warszawskie Zakłady Farmaceutyczne Polfa S.A., Karolkowa, Poland) at 0.5 mg/ml (n=12) or 2 mg/ml (n=12). The vasodilatory effect was measured after 10, 20, 30, 40, 50, and 60 min, as described previously^[12]^. After stabilization, 20 mM KCl was added to the chambers for 10 min to assess the spasm-preventing effect of papaverine. The total amount of tested rings in organ bath were n=24. The AcomPC (Siemens, Germany) software was used in all measurements.

### Immunofluorescence and Morphometry

The method was the same as in the first part of research^[10]^. Twenty arterial rings were immersed for 20 min in phosphate-buffered saline (PBS), in solutions of papaverine 0.5 mg/ml (n=10) or 2 mg/ml (n=10), and then fixed in 4% buffered formalin for 24 hours. After washing and freezing with PBS, 10 µm thick cryostat sections were obtained and the influence of vasodilators on endothelial integrity was studied by immunofluorescence. All 20 rings were serially sectioned and used for immunohistochemical examination at every 20^th^ section. The sections were preincubated with 5% normal goat serum for 40 min, and then were incubated overnight with endothelial cell marker, a mouse monoclonal anti-CD34 antibody (dilution 1:50; Novocastra, Newcastle, UK).

Subsequently, sections were washed extensively in PBS and incubated for 90 min with goat anti-mouse Cy-3-conjugated antibody (dilution 1:400; Jackson IR, West Grove, PA, USA), and then the cell nuclei were counterstained with DAPI (Sigma, Saint Louis, MO, USA). As previously, sections were washed three times in PBS and mounted in glycerol/PBS solution on the same pH=8.6. As in the first part, the endothelial integrity was expressed as the mean percentage of the lumen perimeter immunopositive for CD34.

### Statistical Analysis

Vasodilatory effects of two different papaverine concentrations were checked for normal distribution with the Shapiro-Wilk test. All results obtained by organ bath method and endothelial integrity measurements were expressed as mean ± standard deviation or median (interquartile range; Q1-25^th^ percentile and Q3-75^th^ percentile) unless otherwise stated. To assess the differences between two continuous variables, Student's t-test (for normally distributed values) or the Mann-Whitney U test (for non-normally distributed values) were applied. To assess the differences between three continuous variables, analysis of variance (ANOVA) test (for normally distributed values) or the Kruskal-Wallis test (for non-normally distributed values) were applied.

## RESULTS

**Fig. 1:**
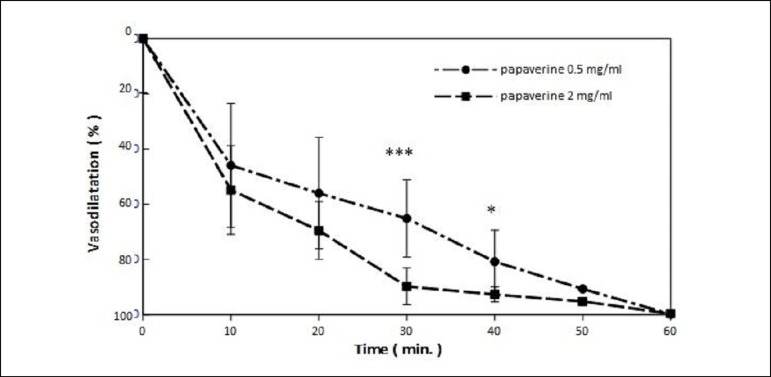
Vasodilatation of potassium chloride (KCl) precontracted radial artery segments induced by papaverine 0.5 mg/ml (n=12) and 2 mg/ml (n=12). Mean±SD. ***P<0.001; SD=standard deviation

The mean vasorelaxation of precontracted RA was stronger in vessel rings incubated in papaverine 2 mg/ml than in 0.5 mg/ml. The statistical significance was reached after 30 min of incubation (papaverine 0.5 mg/ml, 65.19±20.21 *vs.* 2 mg/ml, 89.86±10.45; *P*<0.001; Figure 1). From the practical point of view, it means that the appropriate efficacy is reached after half an hour. The vasorelaxative effect was maintained until the end of vessel examination (60 min).

**Fig. 2:**
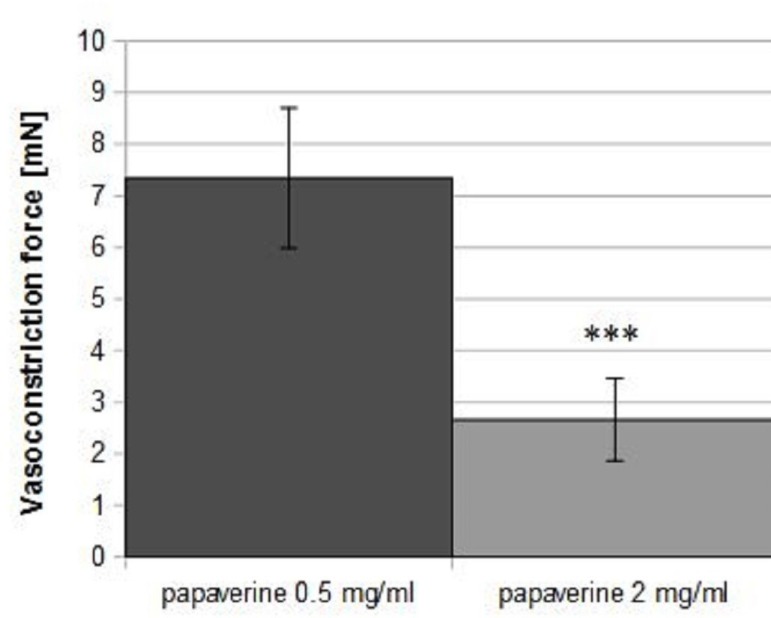
Potassium chloride (KCl) and phenylephrine induced contraction of radial artery segments predilated by papaverine 0.5 mg/ml (n=12) and 2 mg/ml (n=12). ***P=0.083.

The strongest inhibition of vasoconstriction induced by KCl was observed at the highest papaverine dose (2.66±1.96 mN for 2 mg/ml *vs*. 7.35±3.33 mN for 0.5 mg/ml; *P*<0.001; Figure 2).

**Fig. 3:**
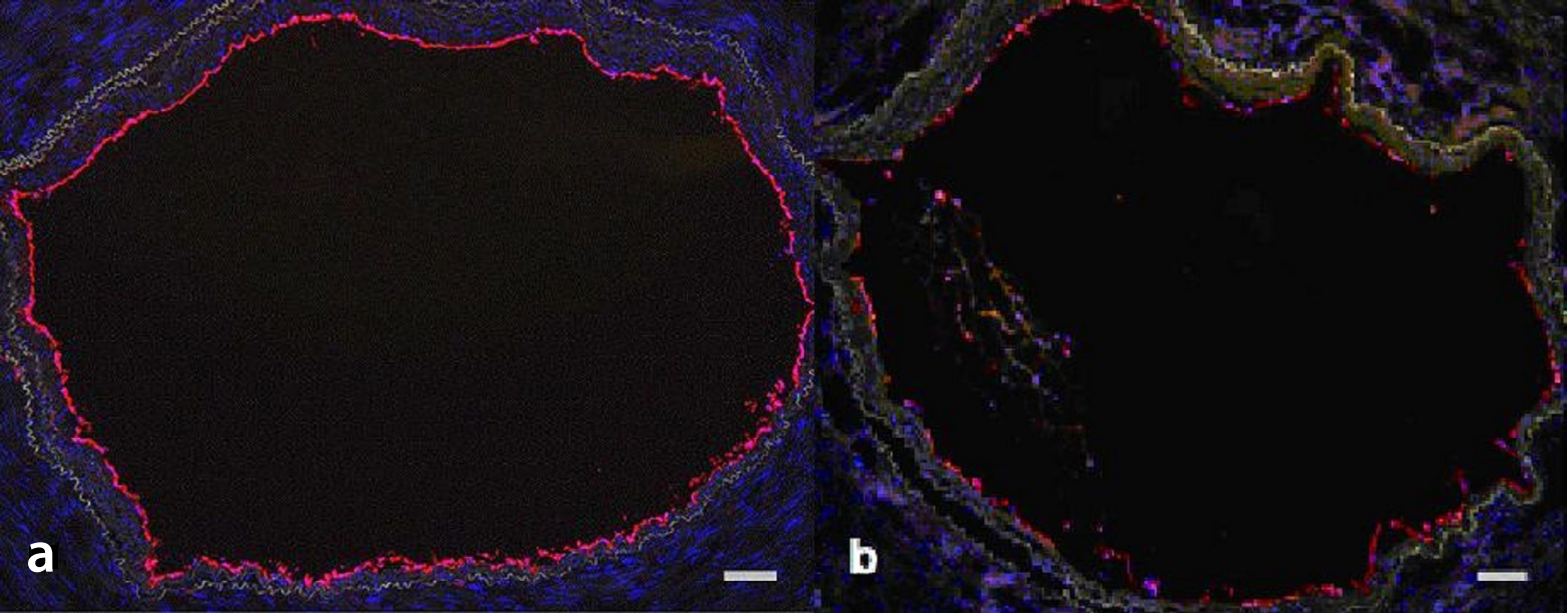
Representative micrographs showing endothelium (red) of radial artery after treatment with papaverine 0.5 mg/ml (a) and 2 mg/ml (b). Note progressive damage to the endothelial lining. Cell nuclei counterstained by DAPI (blue). Bar = 200 µm.

We have also evaluated the impact of the examined papaverine concentration on endothelial integrity by calculating the percentage of vessel's circumference lined by endothelial cells, as revealed by immunofluorescence of their CD34 marker. The endothelial integrity was better preserved after treatment of RA rings with papaverine 0.5 mg/ml (72.86±9.3%) than with papaverine 2 mg/ml (50.23±13.42 %, *P*=0.002) (Figure 3).

## DISCUSSION

Our previous study has shown that when compared to milrinone, papaverine exerts a stronger vasodilatory effect on RA segments and more effectively inhibits their contractile response to KCl and PE. However some studies confirm the dual effect of milrinone and other^[14,15]^ phosphodiesterase (PDE) inhibitors, which reveals an additionally important inotropic effect in medical treatment.

There is no unanimous standpoint concerning papaverine concentration that should be applied in conduits predilatation. In many cardiovascular centers worldwide, different doses of papaverine were examined and applied in predilatation of both RA and internal mammary artery. The efficacy of papaverine was proved for different doses: 0.4 mg/ml^[16,17]^, 1 mg/ml^[18]^, 2.5 mg/ml^[19]^, and 4 mg/ml^[20]^.

At the Department of Cardiovascular Surgery and Transplantology, John Paul II Hospital, where the investigation was carried out, the concentration of papaverine applied for RA predilatation associated with CABG surgery was usually 2 mg/ml.

In terms of vasodilatation and further vasoprotection force, the efficacy of the 0.5 mg/ml papaverine concentration is much lower compared to the 2 mg/ml concentration, and the effects of these two concentrations are comparable; from this viewpoint, both concentrations should be taken under consideration for practical use in CABG surgery. However, papaverine 2 mg/ml caused the highest endothelial damage, at the level of almost 50%. Concentrations of 0.5 mg/ml resulted in much better preservation of endothelial lining.

Immense damage of RA endothelium may lead to undesirable complications during the first postoperative hours in intensive care unit (ICU) because of severe deficiency of nitric oxide (NO), which is synthesized and secreted by endothelial cells. PDE inhibitors increases intracellular cyclic guanosine monophosphate (cGMP) and, as consequence, the level of NO as well^[21]^. It is important to mention that, paradoxically, excess level of PDE concentration causes endothelium destruction and, as a result, lower NO secretion. This might cause a situation when a few hours after CABG surgery, the vasorelaxative and vasoprotective effects of papaverine disappear and the deficiency of NO, one of the most potent vasodilators which is crucial for the regulation of the vascular tone, could lead to a sudden, uncontrolled RA spasm and heart muscle ischemia, creating a direct health hazard with all its consequences for the patient, including low output syndrome or sudden death.

Furthermore, the dysfunction of endothelium induces platelet activation releasing thromboxane A2, which is conductive to thrombosis formation inside the vessel^[21]^, heightening complications in short post-operative period. To elude uncontrolled RA vasospasm, appropriate no-touch harvesting technique and predilatation should be applied. Additionally, it is worth to mention that using RA as a conduit following radial access coronarography is controversial and should be avoided^[22]^.

Thus, taking into consideration the safety (percentage of undamaged endothelium) and our previous research^[10]^, papaverine at concentration 2 mg/ml should not be applied in RA predilatation. The efficacy (maximum vasorelaxation force) of 1 mg/ml examined in the first part of the research^[10]^ is almost identical, but at that concentration papaverine shows significantly better preservation of the endothelium. On the other hand, KCl and PE induced contraction of RA segments predilated by 0.5 mg/ml and 1 mg/ml papaverine is stronger when compared with 2 mg/ml.

## CONCLUSION

Papaverine at 0.5 mg/ml concentration seems to be more suitable than at 2 mg/ml for prevention of vasospasm in RA conduits used for CABG. Papaverine at 2 mg/ml caused the higher endothelial damage. Concentrations of 0.5 mg/ml caused better preservation of the endothelial lining.

**Table:** 

**Authors’ roles & responsibilities**	
PW	Agreement to be accountable for all aspects of the work in ensuring that questions related to the accuracy or integrity of any part of the work are appropriately investigated and resolved; final approval of the version to be published
GL	Agreement to be accountable for all aspects of the work in ensuring that questions related to the accuracy or integrity of any part of the work are appropriately investigated and resolved; final approval of the version to be published
PR	Agreement to be accountable for all aspects of the work in ensuring that questions related to the accuracy or integrity of any part of the work are appropriately investigated and resolved; final approval of the version to be published
JP	Agreement to be accountable for all aspects of the work in ensuring that questions related to the accuracy or integrity of any part of the work are appropriately investigated and resolved; final approval of the version to be published
GPF	Agreement to be accountable for all aspects of the work in ensuring that questions related to the accuracy or integrity of any part of the work are appropriately investigated and resolved; final approval of the version to be published
RK	Agreement to be accountable for all aspects of the work in ensuring that questions related to the accuracy or integrity of any part of the work are appropriately investigated and resolved; final approval of the version to be published
BK	Agreement to be accountable for all aspects of the work in ensuring that questions related to the accuracy or integrity of any part of the work are appropriately investigated and resolved; final approval of the version to be published
KB	Agreement to be accountable for all aspects of the work in ensuring that questions related to the accuracy or integrity of any part of the work are appropriately investigated and resolved; final approval of the version to be published
RL	Agreement to be accountable for all aspects of the work in ensuring that questions related to the accuracy or integrity of any part of the work are appropriately investigated and resolved; final approval of the version to be published

## References

[1] Biancari F, Ruggieri VG, Perrotti A, Svenarud P, Dalén M, Onorati F (2015). European Multicenter Study on Coronary Artery Bypass Grafting
(E-CABG registry): study protocol for a prospective clinical registry and
proposal of classification of postoperative complications. J Cardiothorac Surg.

[2] Litwinowicz R, Bartus K, Drwila R, Kapelak B, Konstanty-Kalandyk J, Sobczynski R (2015). In-hospital mortality in cardiac surgery patients after
readmission to the intensive care unit: a single-center experience with
10,992 patients. J Cardiothorac Vasc Anesth.

[3] Konstanty-Kalandyk J, Piatek J, Rudzinski P, Wrobel K, Bartus K, Sadowski J (2012). Clinical outcome of arterial myocardial revascularization using
bilateral internal thoracic arteries in diabetic patients: a single centre
experience. Interact Cardiovasc Thorac Surg.

[4] Karthik S, Srinivasan AK, Grayson AD, Jackson M, Mediratta NK (2004). Left internal mammary artery to the left anterior descending
artery: effect on morbidity and mortality and reasons for
nonusage. Ann Thorac Surg.

[5] Acar C, Jebara VA, Portoghese M, Beyssen B, Pagny JY, Grare P (1992). Revival of the radial artery for coronary artery bypass
grafting. Ann Thorac Surg.

[6] Tatoulis J, Buxton BF, Fuller JA, Meswani M, Theodore S, Powar N (2009). Long-term patency of 1108 radial arterial-coronary angiograms
over 10 years. Ann Thorac Surg.

[7] Collins P, Webb CM, Chong CF, Moat NE, Radial Artery Versus Saphenous Vein Patency (RSVP) Trial
Investigators (2008). Radial artery versus saphenous vein patency randomized trial:
five-year angiographic follow-up. Circulation.

[8] Ferrari ER, von Segesser LK (2006). Arterial grafting for myocardial revascularization: how better is
it?. Curr Opin Cardiol.

[9] Hayward PA, Buxton BF (2013). Mid-term results of the Radial Artery Patency and Clinical
Outcomes randomized trial. Ann Cardiothorac Surg.

[10] Rudzinski P, Wegrzyn P, Lis GJ, Piatek J, Konstanty-Kalandyk J, Nosalski R (2013). Vasodilatory effect and endothelial integrity in papaverine- and
milrinone-treated human radial arteries. J Physiol Pharmacol.

[11] Mussa S, Guzik TJ, Black E, Dipp MA, Channon KM, Taggart DP (2003). Comparative efficacies and durations of action of
phenoxybenzamine, verapamil/nitroglycerin solution, and papaverine as
topical antispasmodics for radial artery coronary bypass
grafting. J Thorac Cardiovasc Surg.

[12] Guzik TJ, West NE, Pillai R, Taggart DP, Channon KM (2002). Nitric oxide modulates superoxide release and peroxynitrite
formation in human blood vessels. Hypertension.

[13] Shapira OM, Xu A, Aldea GS, Vita JA, Shemin RJ, Keaney Jr JF (1999). Enhanced nitric oxide-mediated vascular relaxation in radial
artery compared with internal mammary artery or saphenous
vein. Circulation.

[14] You Z, Huang L, Cheng X, Wu Q, Jiang X, Wu Y (2016). Effect of milrinone on cardiac functions in patients undergoing
coronary artery bypass graft: a meta-analysis of randomized clinical
trials. Drug Des Devel Ther.

[15] Hernandez-Cascales J (2017). Resveratrol enhances the inotropic effect but inhibits the
proarrhythmic effect of sympathomimetic agents in rat
myocardium. Peer J.

[16] Watanabe G, Yamaguchi S, Takagi T, Tomita S, Tuan PM (2014). Potent vasodilatory effect of fasudil on radial artery graft in
coronary artery bypass operations. Ann Thorac Surg.

[17] Kiessling AH, Romasku D, Beiras-Fernandez A, Ferreirós N, Labocha S, Moritz A (2013). Pharmacokinetics of intraluminally administered serum papaverine
for spasm prophylaxis of the internal mammary artery. Heart Surg Forum.

[18] Rosenfeldt FL, He GW, Buxton BF, Angus JA (1999). Pharmacology of coronary artery bypass grafts. Ann Thorac Surg.

[19] Motallebzadeh R, Wendler O (2005). Reactivity of the human internal thoracic artery to vasodilators
in coronary artery bypass grafting. Eur J Cardiothorac Surg.

[20] Mäyränpää M, Simpanen J, Hess MW, Werkkala K, Kovanen PT (2004). Arterial endothelial denudation by intraluminal use of
papaverine-NaCl solution in coronary bypass surgery. Eur J Cardiothorac Surg.

[21] El-Sayed MI, Amin HA (2015). Mechanism of endothelial cyto-protective and thrombo-resistance
effects of sildenafil, vardenafil and tadalafil in male
rabbit. Arch Med Sci.

[22] Lim LM, Galvin SD, Javid M, Matalanis G (2014). Should the radial artery be used as a bypass graft following
radial access coronary angiography. Interact Cardiovasc Thorac Surg.

